# Supplementary material for: Probing an Ixodes ricinus salivary gland yeast surface display with tick-exposed human sera to identify novel candidates for an anti-tick vaccine
Source: Sci Rep. 2021 Aug 3;11:15745. doi: 10.1038/s41598-021-92538-9 (PMC8333314; doi:10.1038/s41598-021-92538-9)
Supplement: Supplementary file 1 — Supplementary Information 1. [file 41598_2021_92538_MOESM1_ESM.docx]

**Probing an *Ixodes ricinus* salivary gland yeast surface display with tick-exposed human sera to identify novel candidates for an anti-tick vaccine**

Jos J. A. Trentelman^1^, Julen Tomás-Cortázar^2,7*^, Sarah Knorr^3*^, Diego Barriales^2^, Ondrej Hajdusek^4^, Radek Sima^4^, Jasmin I. Ersoz^1^, Sukanya Narasimhan^5^, Erol Fikrig^5^, Ard M. Nijhof^3^, Juan Anguita^2,6^, Joppe W. Hovius^1^.

* both authors contributed equally

*Correspondence to [j.j.trentelman@amsterdamumc.nl](mailto:j.j.trentelman@amsterdamumc.nl)

*Center for Experimental and Molecular Medicine, Amsterdam Multidisciplinary Lyme borreliosis Center, Amsterdam UMC, Location Academic Medical Center, University of Amsterdam, Amsterdam, the Netherlands.^1^; CIC bioGUNE-Basque Research & Technology Alliance, Derio 48160, Spain^2^; Institute for Parasitology and Tropical Veterinary Medicine, Freie Universität Berlin, Berlin, Germany^3^; Biology Centre, Institute of Parasitology, Czech Academy of Sciences, Ceske Budejovice, Czech Republic^4^; Section of Infectious Diseases, Department of Internal Medicine, Yale University, New Haven, CT, USA5^5^; Ikerbasque, Basque Foundation for Science, Bilbao 48012, Spain^6^; UCD Conway Institute, University College Dublin, Belfield, Dublin 4, Ireland^7^*

# Abstract

In Europe, *Ixodes ricinus* is the most important vector of human infectious diseases, most notably Lyme borreliosis and tick-borne encephalitis virus. Multiple non-natural hosts of *I. ricinus* have shown to develop immunity after repeated tick bites. Tick immunity has also been shown to impair *B. burgdorferi* transmission. Most interestingly, multiple tick bites reduced the likelihood of contracting Lyme borreliosis in humans. A vaccine that mimics tick immunity could therefore potentially prevent Lyme borreliosis in humans. A yeast surface display library (YSD) of nymphal *I. ricinus* salivary gland genes expressed at 24, 48 and 72 hours into tick feeding was constructed and probed with antibodies from humans repeatedly bitten by ticks, identifying twelve immunoreactive tick salivary gland proteins (TSGPs). From these, three proteins were selected for vaccination studies. An exploratory vaccination study in cattle showed an anti-tick effect when all three antigens were combined. However, immunization of rabbits did not provide equivalent levels of protection. Our results show that YSD is a powerful tool to identify immunodominant antigens in humans exposed to tick bites, yet vaccination with the three selected TSGPs did not provide protection in the present form. Future efforts will focus on exploring the biological functions of these proteins, consider alternative systems for recombinant protein generation and vaccination platforms and assess the potential of the other identified immunogenic TSGPs.

**Keywords: anti-tick vaccine, yeast surface display, tick salivary gland proteins, *Borrelia*, human, *Ixodes ricinus***

# Background

Vector-borne diseases have an enormous impact on global veterinary and human health. In Europe, *Ixodes ricinus* is the most important vector of human disease^1^. It can harbor and transmit viruses, bacteria and protozoan parasites, which cause a wide range of diseases including tick borne encephalitis, babesiosis, relapsing fever, anaplasmosis and Lyme borreliosis. Lyme borreliosis is the most prevalent tick-borne disease in Europe. Over 65,000 cases of Lyme borreliosis are reported every year throughout Europe, but the incidence could be 2-3 times higher due to underreporting^2^. It is also highly prevalent in the United States, where the main vector for Lyme borreliosis is a closely related *Ixodes* tick species, *I. scapularis*. The causative agent of Lyme borreliosis is a bacterial spirochete of the *Borrelia burgdorferi* sensu lato group, of which *Borrelia afzelii* is the main genospecies causing human disease in Europe and is associated with (chronic) cutaneous manifestations of Lyme borreliosis ^3^.

*I. ricinus* nymphs are considered to be the most clinically relevant life stage with regard to  *B. afzelii* infection in humans, since they are smaller in size and are less easy to notice visually and palplaby^4^. After tick attachment, *B. burgdorferi* s.l. transmission starts within approximately 16-36 hours and experimental evidence from mouse models shows that *B. afzelii*-infected ticks need to feed for longer than 24 hours to establish infection ^5-7^. During feeding *I. ricinus* continuously secretes tick saliva. Tick saliva are important in tick feeding and contains, for example, proteins with anti-complement, anti-hemostatic or immunosuppressive activities ^8-12^, amongst other biologically active proteins. The pharmacological components in tick saliva interfere with processes that are crucial in the host defense response and enable the tick to stay attached to the host and to imbibe blood for days until they are engorged^13^. This is not only beneficial for the tick itself, but also for pathogens that are transmitted by the tick to the host. Indeed, tick saliva has been shown to facilitate infection of the host by these pathogens, also known as saliva assisted transmission (SAT)^14^. SAT has also been shown for *B. burgdorferi* s.l. infection, most notably for *B. burgdorferi* s.s. aided by *I. scapularis* saliva proteins ^8,12,15-17^.

Yet, tick saliva may also be the Achilles’ heel of the tick. Already in 1939 William Trager reported a phenomenon that has been described as tick immunity; repeated infestation with ticks on the same host resulted in reduced number of ticks able to attach and feed, while those that attach are impaired in their ability to feed^18^. Tick immunity has since been described in several animals such as rabbits, guinea pigs and cattle^19-22^. It is a complex and not yet fully elucidated phenomenon in which multiple factors of the immune system, among others T-lymphocytes, eosinophils, mast cells, basophils, complement and importantly antibodies, play a role^22-25^. IgG reacts with tick salivary gland proteins (TSGPs) and increase with multiple infestations^19,26^. Passive transfer of serum from tick immune to naïve animals results in reduced tick infestation as compared to controls, emphasizing that IgG plays an important role in the mechanisms underlying tick immunity^27,28^. Importantly, tick immunity does not only affect tick attachment and feeding, it also affects pathogen transmission. Indeed, tick immune animals are partially protected against *B. burgdorferi* s.s. infection^29,30^ Also here, antibodies play an important role; passive transfer of IgG from animals repeatedly infested with *I. scapularis* nymphs for 24 hours and that developed tick immunity partially protected naive animals against *B. burgdorferi* s.s. infection^31^. Although there is no direct experimental evidence of tick immunity in humans, there are ample anecdotal examples of humans that claim to have developed tick immunity after repeated tick bites^32^. Histological examinations of the tick bite site from repeatedly bitten humans also show inflammatory cell influx compared to more naive individuals indicating at least some form of increased immune activation^33^. In addition, an epidemiological study among 1,489 residents of a Lyme disease endemic area (Block Island, Rhode Island, USA) showed that prior exposure to tick bites associated with redness or itch after a tick bite correlated with a reduced chance of contracting Lyme borreliosis^34^.

Therefore, as part of the current study, we set out to identify *I. ricinus* tick salivary gland proteins recognized by antibodies of repeatedly tick-exposed humans and tested their potential to induce tick immunity. We here describe multiple immunogenic tick saliva proteins identified through a novel yeast surface display expressing *I. ricinus* salivary gland proteins from nymphal ticks, which was probed with total IgG from humans that were repeatedly exposed to tick bites, or controls. In addition, we have assessed the potential of the recombinant forms of selected tick saliva proteins as candidates for an anti-tick vaccine in three different experimental models.

# Results

## **Immunoscreening of a novel *I. ricinus* salivary gland protein - Yeast Surface Display**

A normalized *I. ricinus* salivary gland protein - yeast surface display (SGP-YSD) was created with transcripts of 24, 48 and 72 hours fed nymphs. Human sera from 21 Dutch volunteers (forestry workers) with more than 20 reported tick bites per year were pooled (20TB), as well as sera from five volunteers that reported no tick bites (control) and total IgG was isolated. Western blot analysis using 72 h nymphal salivary gland protein extract showed that 20TB IgG reacted with TSGPs whereas the pooled control did not (Fig. 1a). The 20TB IgG was subsequently used to probe the novel SGP-YSD. First, 3 successive selection rounds were performed using Magnetic-activated cell sorting (MACS) to isolate yeast cells expressing TSGPs recognized by human 20TB IgG increasing the percentage induced SGP-YSD positive cells, for both the expression marker Xpress and 20TB IgG, from 1% to approximately 15% (Fig. 1a). Next, the enriched SGP-YSD was probed with 20TB IgG and fluorescence-activated cell sorting was used to isolate single/clonal induced yeast cells recognized by 20TB IgG and expressing the Xpress tag for sequencing and a sample of the total population for further flow cytometry analysis (Fig. 1b). Flow cytometry analysis showed that the percentage of the induced SGP-YSD positive cells for both the expression marker Xpress and 20TB IgG increased further to approx. 30%. While sorting resulted in 30 times fold enrichment of the SGP-YSD with 20TB IgG, the reactivity with the control serum was only marginally increased (Fig 1a). Finally, a total of 100 isolated single cells were isolated, expanded, PYD1 plasmids isolated, sequenced and a blastx against the UniProt database^35^ was performed to identify the tick proteins encoded and expressed by the respective yeast cell clones.

**Validation of single cell clones and *in silico* analysis of identified tick salivary gland proteins.**

Immunoscreening of the SGP-YSD resulted in the identification of 12 different TSGPs encoded by the 100 single cells that were sequenced (Table 1). Single yeast cell clones for each of the identified proteins were grown, induced and reactivity with 20TB IgG and control IgG was assessed by FACS to confirm differential recognition. When available, multiple clones of each identified TSGP were used for validation. FACS analysis showed that for all clones, reactivity with 20TB IgG was markedly higher than with the control serum, although there was some variation between the identified proteins (Fig. 2). To test nonspecific binding to yeast cell and/or PYD1 expression induced epitopes, a control EBY100 containing an empty PYD1-vector was grown, induced and probed. As expected, only 1% of the cells was positive for 20TB human IgG and no difference in binding of 20TB or control IgG could be observed.

*In silico* analysis of the nucleotide sequences encoded on the plasmids and subsequent amino acid sequences showed that six of the identified TSGPs were full length, either encoded on the plasmid or matched to a full-length sequence from the UniProt database (Table 1). Three of the sequenced clones aligned best with *I. scapularis* proteins (B7PDE7, B7Q4D8, A0A4D5RFW9). Further analysis using the SignalP 4.1 server showed that six proteins, of which four complete sequences (A0A0K8R6W3, V5H126, V5IC40, A0A4D5RFW9) and two incomplete sequences (V5H3P5, V5IGF3), contained a putative signal peptide and were thus predicted to be secreted. B7PDE7 had a very high degree of homology with A0A4D5S8S7 from *I. scapularis* (E-value 0.0, score 1.942, Identity 96.8%), which also had a predicted signal peptide and is therefore also considered to be secreted. Of these seven proteins, two proteins were predicted to also have transmembrane helixes; V5IC40 and A0A4D5RFW9 . Both V5IC40 and A0A4D5RFW9 were considered to be members of the Lamprin protein family, which have a structural role and were located in the extracellular matrix as predicted by the InterPro database.

Interestingly, two identified proteins with complete sequences lacked a signal peptide and had an electron transfer flavoprotein domain (A0A0K8R783) or a ribosome biogenesis regulatory domain (A0A131Y748) as predicted by the InterPro database, making it highly likely that these proteins are located intracellularly. Using the PANTHER classification system it was shown that, except for the putative intracellular proteins A0A0K8R783 and A0A131Y748, all proteins were related to the PANTHER putative-related anchorage subunit family (PTHR33289). This is a small family to which 28 genes from *I. scapularis* have currently been assigned to, together with 17 genes from the marine anemone *Nematostella vectensis.* These TSGPs might be involved in tick cement or a putative other structural function.

**Validation of selected salivary gland proteins**

A0A0K8R6W3, V5H126 and B7PDE7 jointly made up for 72% of the total number of sequenced clones (Table1). Their expression was analyzed with RT-qPCR using RNA from salivary glands of *B. afzelii* infected and uninfected nymphal *I. ricinus* ticks fed for 0, 24 hours and to repletion. A0A0K8R6W3, V5H126 and B7PDE7 are expressed at all time points and no statistical differences could be observed between feeding stages or infection status (Fig 3a-c). More importantly, gene expression analysis showed that all antigens are expressed in all biological replicates of *B. afzelii-*infected and uninfected tick salivary glands in the early feeding stage. They could therefore be potential target candidates for an anti-tick vaccine blocking *Borrelia* transmission. Therefore, recombinant A0A0K8R6W3, V5H126 and B7PDE7 were produced in *E. coli* (Fig. 3d)*.* Interestingly, all three recombinant proteins were recognized by rabbits that acquired tick immunity after repeated challenges with nymphal *I. scapularis* ticks, indicating a possible role in tick immunity. Based on the proportion of identified clones, reactivity of the single cell clones with 20TB sera (Fig. 3e), their expression in the early tick feeding stage and their reactivity with *I. scapularis* tick immune rabbit sera, A0A0K8R6W3, V5H126 and B7PDE7 were selected for exploratory vaccination studies.

**Vaccination experiments**

***Cows***

Cows were vaccinated with PBS as a placebo, or A0A0K8R6W3, V5H126 and B7PDE7 in Montanide ISA 50 V2 with Saponin as adjuvants. For these experiments only 1 cow per experimental group could be used, due to housing limitations. Cows were challenged with 200 nymphal and 100 adult female *I. ricinus* ticks. All experimental groups showed high total IgG levels for their respective antigens as measured by ELISA (Fig 4a-c). Despite high antibody titers, no significant reduction in weights of engorged nymphs was observed during the single antigen immunizations (3.74 ± 1.00 mg, 3.66 ± 0.95 mg, 3.73 ± 1.03 mg vs 3.58 ± 1.02 mg) (Fig. 4d). However, the number of collected fully engorged nymphal ticks were significantly reduced on cows vaccinated with V5H126 (158 vs 180, Chi-Square P<0.01) and A0A0K8R6W3 (147 vs 180, Chi-Square P<0.0001). Vaccination with single antigens did not reduce tick feeding parameters of adult female ticks (Fig 4e). In fact, adult female engorgement weights in the cow vaccinated with A0A0K8R6W3 were slightly, but significantly higher as compared to the control group (272.4 ± 55.9 mg vs 241.5 ± 76.23 mg, one-way ANOVA P<0.05).

In addition, a possible synergistic effect of these three antigens was tested as well, cows were injected with each antigen separately at different injection sites. Vaccination with a combination of A0A0K8R6W3, V5H126 and B7PDE7 did not show an effect either in engorgement weights of nymphs (3.52 ± 0.99 mg vs 3.32 ± 1.06 mg) or in their number (123 vs 109) (Fig. 4f). However, vaccination with all three antigens combined did significantly affect feeding parameters of adult female ticks. The number of collected engorged ticks was significantly reduced (60 vs 77. Chi-Square P<0.01) as well as the mean engorgement weight of the collected ticks (135.1 ± 87.0 mg vs 169.0 ± 89.0 mg) (Fig. 4g). It therefore seems that vaccination with single antigens did not evidently affect tick feeding in cows, but vaccination with all three antigens resulted in a small but significant reduction in the number of engorged adult ticks as well as their engorgement weights, suggesting a synergistical effect.

***Rabbits***

In an attempt to corroborate our findings with the combination of A0A0K8R6W3, V5H126 and B7PDE7 in cows, the vaccinations were repeated in New Zealand white rabbits. Three rabbits per experimental group were again vaccinated with PBS as placebo or A0A0K8R6W3, V5H126 and B7PDE7 in CFA/IFA at separate injection sites. Two weeks after the last vaccination each rabbit was challenged with 50 *I. ricinus* nymphal tick on one ear and 25 female adult ticks on the other. Immunization was successful as indicated by high total IgG titers measured by ELISA (Fig. 5a-c) and vaccination-induced antibodies also reacted with native nymphal and adult salivary gland proteins on Western blot (Fig. 5d). Despite the successful antibody response, no differences in engorgement weights of nymphs were observed (4.17 ± 1.18 mg vs 4.10 ± 1.24 mg) or in the number of nymphs that fed to repletion (41.0 ± 4.6 vs 37.0 ± 3.0) (Fig. 5e). In contrast to the cow vaccination data, vaccination with A0A0K8R6W3, V5H126 and B7PDE7 in rabbits did not result in reduced feeding parameters of adult female ticks. Both the number of engorged ticks (20.7 ± 4.1 vs 23.0 ± 1.0) and their mean weight (207.5 ± 106.1 mg vs 198.8 ± 95.5 mg) were not significantly reduced (Fig. 5f). Thus, the effect of vaccination with A0A0K8R6W3, V5H126 and B7PDE7 on adult tick feeding could not be reproduced in larger experimental groups of rabbits.

**Effect on *B. afzelii* transmission by *I. ricinus* nymphs**

TSGPs are not only important for tick feeding, but can also play a role in SAT and subsequent infection by *Borrelia*. Although vaccination did not result in reduced tick feeding, antibodies against A0A0K8R6W3, V5H126 and B7PDE7 could still be able to affect *B. afzelii* transmission. To this end, mice were passively immunized i.p. with rabbit serum against A0A0K8R6W3, V5H126 and B7PDE7 obtained from our rabbit vaccination experiment. As a control sera from the placebo-vaccinated rabbits were used. Mice were subsequently challenged with eight *B. afzelii-*infected *I. ricinus* nymphs. Passive immunization was successful, as rabbit IgG specific for A0A0K8R6W3 could be detected in mouse plasma by ELISA (Suppl figure 1e.). As expected, passive immunization did not affect the number of engorged nymphs (61 vs 68), or the average engorgement weight (3.57 ± 1.04 mg vs 3.24 ± 1.17 mg) (data not shown). Three weeks after the tick challenge, the mice were sacrificed, skin from the tick bite site, bladder, ankle and heart were collected for *B. afzelii* qPCR, and skin from the tick bite site and bladder were collected for *B. afzelii* culture. Passive immunization with antibodies against A0A0K8R6W3, V5H126 and B7PDE7 did not significantly reduce *B. afzelii* transmission by *I. ricinus* ticks, both the number of infected organs (as determined by qPCR and culture, Table 2) and *Borrelia* loads (determined by PCR, Suppl. Figure 1 a-d) were not statistically different from the control group. Thus, passive immunization with A0A0K8R6W3, V5H126 and B7PDE7 vaccinated rabbit serum could not protect mice against *B. afzelii* transmission from ticks or affect the subsequent course of infection.

**Discussion**

Since the first published description in 1939, there have been multiple reports of tick immunity for multiple tick species and in a plethora of animals ^18,36,37^. Tick immunity not only affects the ability of ticks to attach and/or feed successfully, it may also prevent or reduce successful infection of the host with *B. burgdorferi* s. l. ^27,29-31^. Although there appears to be a cellular component of the immune system involved in tick immunity that should not be overlooked, tick immune animals have developed IgG that recognized TSGPs and passive transfer of serum from tick immune animals also reduces infection of *B. burgdorferi* s.s. by *I. scapularis* nymphs. Antibodies from tick immune animals are therefore exciting tools for antigen discovery, as has been shown previously^31,37^. Interestingly, a survey in a Lyme disease endemic area in the US reported that repeated tick bite exposure reduces the chances of acquiring Lyme disease after a tick bite^34^, providing circumstantial evidence for tick immunity in humans and strengthening the idea for an human anti-tick vaccine. The aim of the current study was to identify *I. ricinus* TSGPs that could be interesting targets for the development of an anti-tick vaccine for humans.

Here we report on the construction an *I. ricinus* SGP-YSD presenting TSGPs expressed at 24, 48 and 72 hours after tick feeding. The YSD technology has previously been successfully used to identify TSGPs in an *I. scapularis* immunoscreening^37^. However, as *B. afzelii* transmission takes place early during tick feeding^5,38-40^, 24 and 48 hour expressed proteins have also been included to the current SGP-YSD resulting in an unprecedented comprehensive expression library^37^. In addition, normalization boosted the prevalence of lowly expressed TSGPs in our library. Although the evidence for tick immunity in humans is largely anecdotal, TSGPs recognized by antibodies from humans with repeated tick bites constitute interesting vaccine candidates and recognized tick proteins are per definition immunogenic in humans. In our study, IgG from human volunteers with more than 20 reported tick bites per year was used to screen the SGP-YSD. Although the species of the ticks responsible for the tick bites in these individuals have not been determined, they are most likely *I. ricinus* ticks as this is by far the most frequently encountered tick species infesting humans in the Netherlands ^41^.

Indeed, immunoscreening of the TSGP-YSD with the selected human IgG proved to be successful. Our initial SGP-YSD library was successfully enriched by MACS and sequencing from sorted single cells led to the identification of 12 immunoreactive TSGPs. Their differential recognition by the screening IgG was confirmed by flow cytometry on respective single cell clones. During the analysis, some variation on the sequence level between multiple clones containing the same protein could be observed. While some sequences were truncated, what could be a result of RNA integrity or splice variants, others showed insertions or deletions mid-sequence at the amino acid level. This could indicate genuine sequence variation, which has been described and reviewed before^42^. It is however doubtful whether the observed variance at the amino acid level could lead to antigenic variance. Only in one of the identified proteins did we observe more than 10% difference in sequence identity between clonal protein sequences. Clonal sequence variation was greatest for A0A0K8R6W3 where the sequence identities varied between 78-100%, however the majority of clones demonstrated 96-100% identity in amino acid sequence (data not shown).

Two proteins were found, A0A0K8R783 and A0A131Y748, that had features that made them highly likely to be intracellular proteins. FACS analysis verified that clonal yeast cells expressing these proteins were indeed recognized by 20TB sera alone, and only upon induction of expression of the PYD1 vector. Therefore, these proteins appear to have entered the tick bite site to be subsequently recognized by the human immune system resulting in specific IgG antibodies. Alternatively, it could be the result of cross-reaction and the human antibodies are directed against a common epitope. It should be noted that is not uncommon to find intracellular tick proteins in immunoscreenings^43^, although the exact mechanism needs to be determined.

The other ten identified proteins belong to the same PANTHER putative-related anchorage subunit family, suggesting some degree of sequence homology. Two of these proteins (V5IC40 and A0A4D5RFW9) also contain a sequence belonging to the lamprin protein family (IPR009437). Lamprin has been first identified in the Sea lamprey and is an insoluble non-collagen, non-elastin protein that is the major component of the fibrillar extracellular matrix of lamprey cartilage. It contains a GGLGY repeated sequence that can be found in insects, for instance in spider dragline silk, or as homologous sequences in mammals and avians. They contain a shared structural motif that has been preserved throughout evolution which self-aggregates and forms fibrils through interdigitation of hydrophobic side chains in beta-sheet/beta-turn structures^44^. These features and the high levels of Glycine (8-18%) in their sequence, might suggest a structural function similar to cement proteins^13,45^. Tick cement is a key element in the tick feeding process: it fills the lateral space between the mouthparts and the skin, forming a cement cone that anchors the tick mouthparts in the host. Although *I. ricinus* re-attaches regularly during feeding and does not seem to produce large cement cones as *Amblyomma hebraeum* ^46,47^, cement proteins are interesting vaccine candidates as demonstrated by 64TRP. 64TRP is a *R. sanguineus* cement protein that has shown to affect tick feeding in multiple species and protect mice against tick-borne encephalitis virus infection from feeding *I. ricinus* ticks ^48-50^.

Based on the fraction of identified single clones and their reactivity with the tick-resistant human sera, A0A0K8R6W3, V5H126 and B7PDE7 were selected to be further investigated as vaccine candidates. Expression analysis showed that these antigens are expressed in the salivary gland of both *B. afzelii-*infected and uninfected nymphal *I. ricinus* in multiple biological replicates*.* Expression levels tended to increase during tick feeding, however due to the large variation in expression levels, this was not statistically significant. Most importantly, all three antigens are present in the early tick feeding stage, which is considered essential for tick feeding and/or *Borrelia* transmission blocking vaccine candidates. Recombinant proteins were made for all three antigens and it was found that these antigens reacted with *I. scapularis* tick immune rabbit sera, validating their possible role in tick immunity.

Cows were vaccinated with the obtained recombinant proteins and challenged with nymphal and female adult *I. ricinus* ticks. Where nymphal ticks are clinically relevant, adult female *I. ricinus* are driving population abundance and are therefore interesting targets for an anti-tick vaccine for wild life^51^. Adult female *I. ricinus* have considerable overlap in their sialome with nymphal ticks and adult female ticks imbibe considerable more blood than nymphal ticks and they also secrete a lot more saliva^52^. We therefore hypothesized that small undetectable effects on engorgement weights in nymphs could lead to a larger detectable effect in adult female ticks due to larger window of measurement and perhaps driven by increased inflammation at the tick bite site due to more saliva. Vaccination led to a significant but small reduction in the number of engorged nymphal ticks for V5H126 and A0A0K8R6W3. Subsequently, cows were vaccinated with a combination of all three antigens and now both the number of engorged adult female ticks as well as their mean engorgement rates were slightly but significantly reduced. To exclude individual variation, vaccination with all three antigens was repeated in rabbits as rabbits are also known to confer strong anti-tick immunity^19,27,31,53^. Although vaccination was successful, vaccination did not show strong tick rejection; no effect on tick parameters for both nymphal and adult ticks could be observed. This could be the result of the increased number of experimental animals used in the rabbit experiment, correcting variances in tick engorgement weights per host. Alternatively, it could be due to more intrinsic differences between the two animal models, i.e. the type of immune responses evoked by vaccination or involved in tick immunity. Or finally, due to the different adjuvants used, although both are proven to assess tick immunity ^54,55^. Although vaccination did not show strong tick rejection, antibodies against these proteins might still be able to inhibit *B. afzelii* transmission, either directly by blocking a possible transmission assisting function of these proteins or indirectly by creating inflammation at the tick bite site hampering successful infection of the host. However, passive immunization of mice with vaccinated rabbit sera did not reduce *B. afzelii* infection after tick challenge (suppl. Fig 1).

A recurring phenomenon in presumed tick immunity in humans is itch at the tick bite site ^32,33,34^. Itch triggers the examination of the affected site and in case of a tick bite site, detection and subsequent removal of the tick. If itch and subsequent tick removal happens early after the tick bite, the risk of pathogen transmission and infection would be greatly reduced. In that light, the resulting itch after a tick bite can be interpreted as a mild form of tick immunity. The tick challenge animal models we used are proven models to assess strong anti-tick rejection. However, the current set-up with contained tick feeding prevents tick removal by scratching of the animals, making it challenging to observe subtle phenotypes as itch and subsequent tick removal. The development of a new challenge model, for instance in humans, that allows the observation of mild phenotypes such as redness and itch would therefore be highly preferable. In addition, there are recent indications that glycosylation of TSGPs contributes to the establishment of tick immunity^36^. Expression of the selected antigens in eukaryotic expression platforms might increase anti-tick efficacy.

In summary, we have established a novel *I. ricinus* SGP-YSD covering nymphal tick proteins expressed at 24, 48 and 72 hours after tick feeding. Using IgG from humans with more than 20 reported tick bites per year we were able to successfully identify as many as 12 immunogenic TSGPs, the majority predicted to be secreted. As part of these studies, three glycine-rich proteins were tested as anti-tick vaccine candidates. Preliminary experiments in cows showing reduced adult tick feeding after vaccination with all three antigens combined, but this could not be repeated in a larger sample size of rabbits. In addition, passive immunization studies did not show an inhibitory effect on *B. afzelli* transmission in mice. While recombinant A0A0K8R6W3, V5H126 and B7PDE7 did not show clear tick rejection using the current experimental setup and strategy, the potential of the other nine TSGPs that are recognized by tick exposed human antibodies still needs to be elucidated. Two proteins appear to be involved in cellular metabolism and the glycine content and PANTHER family for the other ten proteins suggest a possible structural function, but their biological functions need to be experimentally confirmed. The fact that these TSGPs are recognized by antibodies from tick exposed humans make them not only interesting vaccine candidates, but also studying the function of these proteins could increase our insight into the tick-human interactions that shape the feeding process and transmission of pathogens from the tick to the host.

**Material and methods**

**Construction of a nymphal *I. ricinus* salivary gland cDNA library and subsequent yeast surface display**

Nymphal *I. ricinus* ticks were fed on rabbits for 24 (800 nymphs), 48 (400 nymphs) and 72 hours (150 nymphs). After removal from the rabbit, salivary glands were isolated, pooled, lysed in ML buffer (MACHEREY-NAGEL GmbH & Co KG) and stored at -80°C. Small and large RNA was extracted using the miRNA kit (MACHEREY-NAGEL GmbH & Co KG) for each time point and pooled. cDNAs were prepared and directionally cloned into the EcoRI and NotI sites of the yeast expression vector pYD1 (Invitrogen, CA). The subsequent cDNA library was normalized to Cot40. PstI digestion of plasmids of the pYD1-salivary gland library, showed an average insert size of 1.5 kb and 100% of the clones contained inserts. The unamplified library titre was 2,8x10^6^ cfu/ml. Yeast cells were transformed by electroporation as described by Chao et al^56^. Briefly, fresh *Saccharomyces cerevisiae* EBY100 cells (ATCC, Manassas, Virginia, USA) were electroporated together with 5 µg of DNA and subsequently grown in SDCAA medium (2% dextrose, 0.67% yeast nitrogen base, 0.5% bacto amino acids, 30 mM NaHPO4, 62 mM NaH2PO4) at 30 °C.

**Human sera selection and IgG purification**

Previously collected sera of 21 human volunteers that had more than 20 reported tick bites per year was pooled (20TB)^57,58^. Five of the volunteers also reported to develop itch after each tick bite. As a control, sera from five human volunteers with no reported tick bites was pooled. From both pools total IgG was purified using the Melon Gel IgG purification kit (Thermo scientific, Waltham, Massachusetts, USA). Total protein concentration was measured using the Pierce BCA protein assay kit (Thermo Scientific, Waltham, Massachusetts, USA). Quality of the IgG was checked on a 4-20% BIS-TRIS gel by SDS page under nun-reducing conditions.

**Immunoscreening of the obtained yeast surface display**

1x 10^9^ transformed yeast cells were grown in SDCAA for 24 hours at 30 °C. These freshly grown yeast cells were induced overnight in 100 ml SGCAA medium (2% w/v galactose, 0.67% w/v yeast nitrogen base, 0.5% w/v bacto amino acids, 30 mM NaHPO4, 62 mM NaH2PO4), 30 °C at an initial OD of 0.6. 4x10^8^ induced cells were washed 3 times with PBSM+B buffer (5 g/L bovine serum albumin and 2 mM EDTA in PBS). The induced yeast cells were subsequently incubated with 50 µg/ml of purified 20TB IgG in a rotator for 15 minutes at room temperature and 45 minutes at 4°C. Cells were washed 3 times with PBSM+B and incubated with Anti-IgG MicroBeads, human (Miltenyi Biotech, Germany) for 20 minutes at 4°C. Cells were again washed 3 times and were loaded on a MS column (Miltenyi Biotech, Germany) in a MiniMACS™ Separator (Miltenyi Biotech, Germany). The column was washed three times with PBSM buffer, removed from the separator and eluted with SDCAA medium. The collected yeast cells were expended in SDCAA overnight. This process was repeated 3 times. After the last MACS sort, single cells were sorted by FACS. Yeast cells were grown and induced as before and washed times with FACSsort buffer (5g/L bovine serum albumin, 0.35 mM EDTA). Induced cells were labeled with 50 µg/ml human IgG (control or 20TB) and 1:500 mouse anti-Xpress IgG in a rotator for 15 minutes at room temperature and 45 minutes at 4°C. Cells were washed 3 times with FACSsort buffer and incubated with anti-IgG-FITC human (50 µg/ml) and anti-IgG1-APC-Vio mouse (Biotech, Germany) for 60 minutes at 4°C. Induced yeast cells incubated with control human serum was used to set the sorting gate. Single induced yeast cells differentially recognized by 20TB IgG were sorted on a Sony SH800 in 96 well cell culture plates (Greiner Bio-one, Kremsmünster, Austria) containing SDCAA medium. Plates were subsequently grown overnight at 30°C. From 100 isolated clones, PYD-1 plasmids were isolated with Zymoprep Yeast Plasmid Miniprep II (Zymo Research, Irvine, California, USA), according to the manufacturers protocol. The obtained plasmids were sequenced using the primers shown in supplemental file 1 and blasted against the Uniprot database.

**FACS analysis**

Freshly grown yeast cells were induced overnight in SGCAA medium, 30 °C at an initial OD of 0.6. 1x10^6^ induced cells were washed 3 times with FACS buffer (5 g/L bovine serum albumin, 0.35 mM EDTA, 0.01% w/v NaN_3_ in PBS). The induced yeast cells were subsequently incubated with 50 µg/ml of purified 20TB IgG and 1:500 Xpress monoclonal antibody (Invitrogen, California, USA) in a rotator for 15 minutes at room temperature and 45 minutes at 4°C. Cells were washed 3 times with FACS buffer and incubated with anti-IgG-FITC human (Southern biotech, 1:100) and anti-IgG1-APC-Vio mouse (Miltenyi Biotech, Germany ,1:50) for 1 hour at 4°C. Cells were again washed 3 times and were loaded on a MS column (Miltenyi Biotech, Germany) in a MiniMACS™ Separator (Miltenyi Biotech, Germany). The column was washed three times with PBSM buffer, removed from the separator and fixed with 4% w/v paraformaldehyde in PBS for 20 min on ice. Cells were spin down and resuspended in FACS buffer for FACS analysis on a FACSCalibur (BD, San Jose, CA).

**Gene expression profiles**

Gene expression profiles were assessed as before^59^. *Borrelia afzelii*-infected (Infection rates were assessed by qPCR and ticks were used when infection rates were higher than 90%) and uninfected nymphal *I. ricinus* ticks derived from 10 distinct egg batches laid by adult female ticks collected from the wild, were fed on mice for different time points. RNA was isolated from the salivary glands and subsequent cDNA was prepared for the individual time points. Then, gene-specific primers were designed using Primer3 software (Supplemental file 1) and used in qRT-PCR to evaluate expression of the selected genes. The qPCR reactions were performed in triplicate using LightCycler480 (Roche, Nutley, NJ, USA), SensiFAST SYBR No-ROX mix (Bioline GmbH, Germany) and the protocol used was 95 °C 6 min, and 60 cycles of 95 °C 10 s, 60 °C 20 s and 72 °C 20 s.

**Expression and purification of recombinant proteins**

TSGPs were cloned by overlapping PCR from the isolated PYD1 plasmids and cloned as NcoI-SalI fragments into the pHIS-parallel 2 expression vector^60^. Clones were induced with 1 mM Isopropyl-β-D-thiogalactoside (IPTG) for 16 h at 20°C in E. coli BL21 C41(DE3). The bacterial cells were then lysed and centrifuged. The expressed insoluble proteins were extracted from the inclusion bodies with the following protocol. The pellets were thoroughly homogenized in Phosphate Buffered Saline (PBS); 2% Triton X-100 followed by an incubation at 37°C for 30 minutes with shaking. The samples were ultracentrifuged at 96,000 g for 30 min and the pellets were homogenized again in PBS and incubated at 37°C for 30 minutes with shaking. After a second ultracentrifugation, the pellets were homogenized in PBS; 7M urea. The denatured proteins were dialyzed to 2M urea overnight.

**Cow vaccinations**

Single cows were vaccinated twice with either PBS, V5H126, B7PDE7, A0A0K8R6W3 or, in a second experiment, with PBS or all three antigens combined with a six-week interval. For every vaccination 100 µg of each recombinant protein was emulsified with 1.5 mg saponin in 1 mL of Montanide ISA V50 adjuvant (SEPPIC, France) as specified by the adjuvant’s manufacturer. Two weeks after the last vaccination, cows were challenged as described previously^61^: Two weeks after the second immunization, each animal was infested with 200 nymphs, 100 adult males, and 100 *I. ricinus* females. The ticks were equally divided over two linen bags, which were attached to the basis of the unshaved ears using adhesive tape (Leukoplast, BSN medical, Hamburg, Germany). Following a resting period of 2 days to allow ticks to attach undisturbed, bags were checked twice per day and engorged nymphs or females were removed and weighed. Adult females were subsequently stored individually and nymphs were stored in small batches in glass tubes. The tubes containing the ticks were stored in a desiccator with 90% relative humidity (RH), which was placed in a climate chamber at 20°C and a light-dark-cycle of 14–10 h.

**Rabbit vaccinations**

Rabbits were vaccinated as described before^62^. In short, New Zealand white rabbits (Charles River Laboratories) were vaccinated with 50 µg of V5H126, B7PDE7 and A0A0K8R6W3 in complete Freund’s adjuvant followed by two vaccinations with 50 µg of V5H126, B7PDE7 and A0A0K8R6W3 in incomplete Freund’s adjuvant with three weeks interval. Two weeks after the last vaccination, the rabbits were challenged with 25 adult female and 25 adult male *I. ricinus* on one ear and 50 nymphal *I. ricinus* on the other ear. Ticks placed on the ears were contained by cotton socks taped to the ears and a neck collar prevented manipulation by the rabbit (soft eCollar, MDC exports, UK). The ticks were checked daily and dropped off ticks were weighted and stored in a desiccator with 80% relative humidity (RH), which was placed in a climate chamber at 20°C and a light-dark-cycle of 14–10 h.

**Passive immunizations**

Eight C3H/HeN mice were passively immunized i.p. with 200 µl serum from rabbits vaccinated with V5H126, B7PDE7 and A0A0K8R6W3 or PBS. 24 hours after passive immunization, the mice were challenged with eight *B. afzelii* infected *I. ricinus* nymphs. Three weeks after infestation, mice were sacrificed and organs collected for detection of *B. afzelii*. Skin from the tick bite site and bladder were used for culturein modified Kelly Pettenkofer (MKP) medium with rifampicin, 50 µg/ml and phosphomycin, 100 µg/ml) at 33 °C. The cultures were checked weekly with dark field microscopy as described before^63^. DNA was isolated from skin from the tick bite site, bladder, heart and joint using the Blood and tissue kit (Qiagen, Venlo, The Netherlands) and *B. afzelii* DNA quantified by qPCR as described previously ^64^. Primers can be found in Supplemental file 1.

**Antigen specific antibody responses**

Total antigen-specific IgG levels were determined by ELISA. 96 high-binding half area microtiter plates (Greiner Bio-one, Kremsmünster, Austria) were coated with recombinant proteins at 25 ng/well in carbonate buffer (pH 9.6) and incubated overnight RT. After washing with wash buffer (PBS, containing 0.05% Tween 20), the plates were incubated with blocking buffer (1% fetal calf serum (FCS, Biowest) in PBS) for 1 h. Sera were diluted in blocking buffer and incubated for 1 h at room temperature. After washing, HRP conjugated goat anti-mouse IgG (Cell Signaling, Beverly, MA, USA) or HRP conjugated goat anti-rabbit IgG (Cell Signaling, Beverly, MA, USA) was added in blocking buffer and incubated for 1 h at room temperature. The plates were then extensively washed and incubated with TMB as substrate (Sigma-Aldrich, MO, USA . The reaction was stopped with 2N H_2_SO_4_. Absorbance (450-650 nm) was immediately measured using a BioTek Synergy HT multi-detection microplate reader. A similar ELISA protocol was used for cow antibody titers as described previously^61^.

**Ethical Statement**

All animal experiments were performed in accordance with relevant guidelines and regulations and the ARRIVE guidelines. All mouse and rabbit vaccination studies were conducted with approval of the commission for animal experiments (DEC AMC, Amsterdam, under CCD license AVD1180020171785). All cattle vaccination experiment were conducted with approval of the commission for animal experiments (LAGeSo, Berlin, registration number G0210/15). Human sera was collected in 1989 after informed consent was obtained from all subjects as described previously^57,58^ and in accordance with relevant guidelines and regulations of the AMC and the WMA Declaration of Helsinki. The use of these sera for the experimental protocols described here were approved by the IRB of the AMC.

**Acknowledgements**

This project has received funding from the European Union’s Seventh Programme for research, technological development and demonstration under grant agreement No. 602272 of which JWH was the scientific coordinator. This work was also supported by a “Vidi” grant (09150171910024) from JWH received from The Netherlands Organisation for health research and development (ZonMw). AMN received financial support by the Federal Ministry of Education and Research (BMBF) under project number 01KI1720 as part of the ‘Research Network Zoonotic Infectious Diseases.’ The following reagents was provided by Centers for Disease Control and Prevention for distribution by BEI Resources, NIAID, NIH: *Ixodes ricinus* Nymph (Live), NR-44118 and *Ixodes ricinus* Adult (Live), NR-42511.

**Author Contributions**

JWH was the scientific coordinator. JT performed YSD screening, FACS analysis, gene expression analysis and *in silico* analysis. RS and OH provided ticks and tick salivary gland RNA. JTC, DB and JA produced recombinant proteins. SK, JE, JT, AN, JA and JWH were involved in vaccination experiments. RS, OH, SN, EF, AN, JA, SK, JT and JWH designed the experiments. JT and JWH wrote the manuscript with input from all authors.

**Corresponding author**

Correspondence to Jos J.A. Trentelman

**Additional Information**

The author(s) declare no competing interests.

**Table 1. see .ppt**

Tick salivary gland proteins identified. Nucleotide sequences from the best clones for each identified TSGP were blasted against the UniProt database^35^ resulting in identification of the TSGPs and subsequent Identity, E value, Score and protein status. Signal peptides were predicted based on amino acid sequence by SignalP 5.0 server^65^ and HTHMM v2.0 server^66^ was used to predict transmembrane helices. Proteins sequences were subsequently scanned for domains with InterProScan^67^.

**Table 2. Number of *Borrelia afzelii*-infected mice as determined for each organ.**

|  | **Skin^1^** | **Bladder^1^** | **Skin^2^** | **Bladder^2^** | **Heart^2^** | **Joint^2^** |
| --- | --- | --- | --- | --- | --- | --- |
| **Anti-PBS** | 5/8 | 6/8 | 8/8 | 7/8 | 5/8 | 7/8 |
| **Anti-A0A0K8R6W3, V5H126, B7PDE7** | 5/8 | 5/8 | 8/8 | 7/8 | 6/8 | 6/8 |

*Borrelia afzelii* infection as determined by culture^1^ or qPCR^2^ and shown as number of positive mice/total mice.

# References

1 Semenza, J. C. & Suk, J. E. Vector-borne diseases and climate change: a European perspective. *FEMS Microbiology Letters* **365**, doi:10.1093/femsle/fnx244 (2017).

2 Hubálek, Z. Epidemiology of lyme borreliosis. *Curr Probl Dermatol* **37**, 31-50, doi:10.1159/000213069 (2009).

3 van Dam, A. P. *et al.* Different genospecies of Borrelia burgdorferi are associated with distinct clinical manifestations of Lyme borreliosis. *Clin Infect Dis* **17**, 708-717, doi:10.1093/clinids/17.4.708 (1993).

4 Embers, M. E. & Narasimhan, S. Vaccination against Lyme disease: past, present, and future. *Frontiers in cellular and infection microbiology* **3**, 6-6, doi:10.3389/fcimb.2013.00006 (2013).

5 Crippa, M., Rais, O. & Gern, L. Investigations on the Mode and Dynamics of Transmission and Infectivity of Borrelia burgdorferi Sensu Stricto and Borrelia afzelii in Ixodes ricinus Ticks. *Vector-Borne and Zoonotic Diseases* **2**, 3-9, doi:10.1089/153036602760260724 (2002).

6 Pospisilova, T. *et al.* Tracking Borrelia afzelii from infected Ixodes ricinus nymphs to mice suggests a direct 'gut-to-mouth' route of Lyme disease transmission. 316927, doi:10.1101/316927 %J bioRxiv (2018).

7 Cook, M. J. Lyme borreliosis: a review of data on transmission time after tick attachment. *Int J Gen Med* **8**, 1-8, doi:10.2147/IJGM.S73791 (2014).

8 Anguita, J. *et al.* Salp15, an ixodes scapularis salivary protein, inhibits CD4(+) T cell activation. *Immunity* **16**, 849-859 (2002).

9 Chmelar, J., Kotal, J., Kovarikova, A. & Kotsyfakis, M. The Use of Tick Salivary Proteins as Novel Therapeutics. *Frontiers in physiology* **10**, 812, doi:10.3389/fphys.2019.00812 (2019).

10 Hovius, J. W. *et al.* Preferential Protection of Borrelia burgdorferi Sensu Stricto by a Salp 15 Homologue in Ixodes ricinus Saliva. *Journal of Infectious Diseases* **198**, 1189-1197, doi:10.1086/591917 (2008).

11 Schuijt, T. J. *et al.* Factor Xa Activation of Factor V is of Paramount Importance in Initiating the Coagulation System: Lessons from a Tick Salivary Protein. *Circulation* **128**, 10.1161/CIRCULATIONAHA.1113.003191, doi:10.1161/CIRCULATIONAHA.113.003191 (2013).

12 Wagemakers, A. *et al.* An Ixodes ricinus Tick Salivary Lectin Pathway Inhibitor Protects Borrelia burgdorferi sensu lato from Human Complement. (2016).

13 Francischetti, I. M., Sa-Nunes, A., Mans, B. J., Santos, I. M. & Ribeiro, J. M. The role of saliva in tick feeding. *Frontiers in bioscience (Landmark edition)* **14**, 2051-2088, doi:10.2741/3363 (2009).

14 Nuttall, P. A. & Labuda, M. Tick-host interactions: saliva-activated transmission. *Parasitology* **129 Suppl**, S177-189, doi:10.1017/s0031182004005633 (2004).

15 Dai, J. *et al.* Tick Histamine Release Factor Is Critical for *Ixodes scapularis* Engorgement and Transmission of the Lyme Disease Agent. *PLoS pathogens* **6**, e1001205, doi:10.1371/journal.ppat.1001205 (2010).

16 Dai, J. *et al.* Antibodies against a tick protein, Salp15, protect mice from the Lyme disease agent. *Cell Host Microbe* **6**, 482-492, doi:10.1016/j.chom.2009.10.006 (2009).

17 Schuijt, T. J. *et al.* A tick mannose-binding lectin inhibitor interferes with the vertebrate complement cascade to enhance transmission of the lyme disease agent. *Cell Host Microbe* **10**, 136-146, doi:10.1016/j.chom.2011.06.010 (2011).

18 Trager, W. Acquired Immunity to Ticks. *The Journal of Parasitology* **25**, 57-81, doi:10.2307/3272160 (1939).

19 Bowessidjaou, J., Brossard, M. & Aeschlimann, A. Effects and duration of resistance acquired by rabbits on feeding and egg laying in Ixodes ricinus L. *Experientia* **33**, 528-530, doi:10.1007/BF01922254 (1977).

20 Mbow, M. L., Christe, M., Rutti, B. & Brossard, M. ABSENCE OF ACQUIRED-RESISTANCE TO NYMPHAL IXODES-RICINUS TICKS IN BALB/C MICE DEVELOPING CUTANEOUS REACTIONS. *Journal of Parasitology* **80**, 81-87, doi:10.2307/3283349 (1994).

21 Ribeiro, J. M. C. ROLE OF SALIVA IN TICK HOST INTERACTIONS. *Experimental & Applied Acarology* **7**, 15-20, doi:10.1007/bf01200449 (1989).

22 Wada, T. *et al.* Selective ablation of basophils in mice reveals their nonredundant role in acquired immunity against ticks. *Journal of Clinical Investigation* **120**, 2867-2875, doi:10.1172/jci42680 (2010).

23 Brossard, M. & Wikel, S. K. Immunology of interactions between ticks and hosts. *Medical and Veterinary Entomology* **11**, 270-276, doi:10.1111/j.1365-2915.1997.tb00406.x (1997).

24 Brossard, M. & Wikel, S. K. in *Parasitology* Vol. 129 S161-S176 (2004).

25 Wikel, S. K. Host immunity to ticks. *Annual review of entomology* **41**, 1-22 (1996).

26 Lawrie, C. H. & Nuttall, P. A. Antigenic profile of Ixodes ricinus: effect of developmental stage, feeding time and the response of different host species. *Parasite Immunology* **23**, 549-556, doi:10.1046/j.1365-3024.2001.00412.x (2001).

27 Brossard, M. & Girardin, P. Passive transfer of resistance in rabbits infested with adult Ixodes ricinus L: humoral factors influence feeding and egg laying. *Experientia* **35**, 1395-1397 (1979).

28 Roberts, J. A. & Kerr, J. D. Boophilus microplus: passive transfer of resistance in cattle. *J Parasitol* **62**, 485-488 (1976).

29 Nazario, S. *et al.* Prevention of Borrelia burgdorferi transmission in guinea pigs by tick immunity. *The American journal of tropical medicine and hygiene* **58**, 780-785 (1998).

30 Wikel, S. K., Ramachandra, R. N., Bergman, D. K., Burkot, T. R. & Piesman, J. Infestation with pathogen-free nymphs of the tick Ixodes scapularis induces host resistance to transmission of Borrelia burgdorferi by ticks. *Infection and Immunity* **65**, 335-338 (1997).

31 Narasimhan, S. *et al.* Immunity against *Ixodes scapularis* Salivary Proteins Expressed within 24 Hours of Attachment Thwarts Tick Feeding and Impairs *Borrelia* Transmission. *PLoS ONE* **2**, e451, doi:10.1371/journal.pone.0000451 (2007).

32 Moyer, M. W. The growing global battle against blood-sucking ticks. *Nature* **524**, 406-408, doi:10.1038/524406a (2015).

33 Krause, P. J. *et al.* Dermatologic changes induced by repeated Ixodes scapularis bites and implications for prevention of tick-borne infection. *Vector borne and zoonotic diseases (Larchmont, N.Y.)* **9**, 603-610, doi:10.1089/vbz.2008.0091 (2009).

34 Burke, G. S. *et al.* Hypersensitivity to ticks and Lyme disease risk. *Emerging Infectious Diseases* **11**, 36-41 (2005).

35 Consortium, T. U. UniProt: a worldwide hub of protein knowledge. *Nucleic Acids Research* **47**, D506-D515, doi:10.1093/nar/gky1049 (2018).

36 Narasimhan, S. *et al.* Ixodes scapularis saliva components that elicit responses associated with acquired tick-resistance. *Ticks and Tick-borne Diseases*, 101369, doi:<https://doi.org/10.1016/j.ttbdis.2019.101369> (2020).

37 Schuijt, T. J. *et al.* Identification and characterization of Ixodes scapularis antigens that elicit tick immunity using yeast surface display. *PLoS One* **6**, e15926, doi:10.1371/journal.pone.0015926 (2011).

38 Pospisilova, T. *et al.* Tracking of Borrelia afzelii transmission from infected Ixodes ricinus nymphs to mice. *Infection and Immunity*, IAI.00896-00818, doi:10.1128/IAI.00896-18 (2019).

39 Crippa, M., Rais, O. & Gern, L. Investigations on the mode and dynamics of transmission and infectivity of Borrelia burgdorferi sensu stricto and Borrelia afzelii in Ixodes ricinus ticks. *Vector borne and zoonotic diseases (Larchmont, N.Y.)* **2**, 3-9, doi:10.1089/153036602760260724 (2002).

40 Piesman, J., Mather, T. N., Sinsky, R. J. & Spielman, A. Duration of tick attachment and Borrelia burgdorferi transmission. *Journal of Clinical Microbiology* **25**, 557-558 (1987).

41 Hofhuis, A. *et al.* Decrease in tick bite consultations and stabilization of early Lyme borreliosis in the Netherlands in 2014 after 15 years of continuous increase. *BMC Public Health* **16**, 425, doi:10.1186/s12889-016-3105-y (2016).

42 Chmelař, J., Kotál, J., Kopecký, J., Pedra, J. H. F. & Kotsyfakis, M. All For One and One For All on the Tick-Host Battlefield. *Trends Parasitol* **32**, 368-377, doi:10.1016/j.pt.2016.01.004 (2016).

43 Lewis, L. A., Radulović, Ž. M., Kim, T. K., Porter, L. M. & Mulenga, A. Identification of 24h Ixodes scapularis immunogenic tick saliva proteins. *Ticks and Tick-borne Diseases* **6**, 424-434, doi:<https://doi.org/10.1016/j.ttbdis.2015.03.012> (2015).

44 Robson, P. *et al.* Characterization of lamprin, an unusual matrix protein from lamprey cartilage. Implications for evolution, structure, and assembly of elastin and other fibrillar proteins. *Journal of Biological Chemistry* **268**, 1440-1447 (1993).

45 Suppan, J., Engel, B., Marchetti-Deschmann, M. & Nürnberger, S. Tick attachment cement – reviewing the mysteries of a biological skin plug system. *Biological Reviews* **93**, 1056-1076, doi:10.1111/brv.12384 (2018).

46 Krober, T. & Guerin, P. M. In vitro feeding assays for hard ticks. *Trends Parasitol* **23**, 445-449, doi:10.1016/j.pt.2007.07.010 (2007).

47 Krober, T. & Guerin, P. M. An in vitro feeding assay to test acaricides for control of hard ticks. *Pest Manag Sci* **63**, 17-22, doi:10.1002/ps.1293 (2007).

48 Labuda, M. *et al.* An Antivector Vaccine Protects against a Lethal Vector-Borne Pathogen. *PLoS pathogens* **2**, e27, doi:10.1371/journal.ppat.0020027 (2006).

49 Trimnell, A. R., Davies, G. M., Lissina, O., Hails, R. S. & Nuttall, P. A. A cross-reactive tick cement antigen is a candidate broad-spectrum tick vaccine. *Vaccine* **23**, 4329-4341, doi:10.1016/j.vaccine.2005.03.041 (2005).

50 Trimnell, A. R., Hails, R. S. & Nuttall, P. A. Dual action ectoparasite vaccine targeting 'exposed' and 'concealed' antigens. *Vaccine* **20**, 3560-3568, doi:10.1016/s0264-410x(02)00334-1 (2002).

51 Hofmeester, T. R., Sprong, H., Jansen, P. A., Prins, H. H. T. & van Wieren, S. E. Deer presence rather than abundance determines the population density of the sheep tick, Ixodes ricinus, in Dutch forests. *Parasites & Vectors* **10**, 433, doi:10.1186/s13071-017-2370-7 (2017).

52 Schwarz, A. *et al.* A systems level analysis reveals transcriptomic and proteomic complexity in Ixodes ricinus midgut and salivary glands during early attachment and feeding. *Mol Cell Proteomics* **13**, 2725-2735, doi:10.1074/mcp.M114.039289 (2014).

53 Sprong, H. *et al.* ANTIDote: anti-tick vaccines to prevent tick-borne diseases in Europe. *Parasit Vectors* **7**, doi:10.1186/1756-3305-7-77 (2014).

54 Mulenga, A. *et al.* Molecular Characterization of a &lt;em&gt;Haemaphysalis longicornis&lt;/em&gt; Tick Salivary Gland-Associated 29-Kilodalton Protein and Its Effect as a Vaccine against Tick Infestation in Rabbits. *Infection and Immunity* **67**, 1652, doi:10.1128/IAI.67.4.1652-1658.1999 (1999).

55 Raman, P. M., Ramadass, P. & Rajavelu, G. Evaluation of Saponin and Montanide ISA 50 adjuvants for their immunopotency and effect on humoral immune response of calves to purified midgut antigen of Boophilus microplus. *Veterinarski Arhiv* **74**, 129-140 (2004).

56 Chao, G. *et al.* Isolating and engineering human antibodies using yeast surface display (vol 1, pg 755, 2006). *Nature Protocols* **1** (2006).

57 Kuiper, H. *et al.* Lyme borreliosis in dutch forestry workers. *Journal of Infection* **23**, 279-286, doi:<https://doi.org/10.1016/0163-4453(91)92936-Y> (1991).

58 Kuiper, H., van Dam, A. P., Moll van Charante, A. W., Nauta, N. P. & Dankert, J. One year follow-up study to assess the prevalence and incidence of Lyme borreliosis among Dutch forestry workers. *Eur J Clin Microbiol Infect Dis* **12**, 413-418, doi:10.1007/bf01967434 (1993).

59 Trentelman, J. J. A. *et al.* A combined transcriptomic approach to identify candidates for an anti-tick vaccine blocking B. afzelii transmission. *Sci Rep* **10**, 20061, doi:10.1038/s41598-020-76268-y (2020).

60 Sheffield, P., Garrard, S. & Derewenda, Z. Overcoming expression and purification problems of RhoGDI using a family of "parallel" expression vectors. *Protein Expr Purif* **15**, 34-39, doi:10.1006/prep.1998.1003 (1999).

61 Knorr, S. *et al.* Preliminary Evaluation of Tick Protein Extracts and Recombinant Ferritin 2 as Anti-tick Vaccines Targeting Ixodes ricinus in Cattle. *Frontiers in physiology* **9**, doi:10.3389/fphys.2018.01696 (2018).

62 Coumou, J., Wagemakers, A., Trentelman, J. J., Nijhof, A. M. & Hovius, J. W. Vaccination against Bm86 Homologues in Rabbits Does Not Impair Ixodes ricinus Feeding or Oviposition. *PLoS One* **10**, e0123495, doi:10.1371/journal.pone.0123495 (2014).

63 Klouwens, M. J. *et al.* Investigating BB0405 as a novel Borrelia afzelii vaccination candidate in Lyme borreliosis. *Sci Rep* **11**, 4775, doi:10.1038/s41598-021-84130-y (2021).

64 Wagemakers, A. *et al.* Rapid outer-surface protein C DNA tattoo vaccination protects against Borrelia afzelii infection. *Gene Therapy* **21**, 1051-1057, doi:10.1038/gt.2014.87 (2014).

65 Almagro Armenteros, J. J. *et al.* SignalP 5.0 improves signal peptide predictions using deep neural networks. *Nature Biotechnology* **37**, 420-423, doi:10.1038/s41587-019-0036-z (2019).

66 Krogh, A., Larsson, B., von Heijne, G. & Sonnhammer, E. L. L. Predicting transmembrane protein topology with a hidden markov model: application to complete genomes11Edited by F. Cohen. *Journal of Molecular Biology* **305**, 567-580, doi:<https://doi.org/10.1006/jmbi.2000.4315> (2001).

67 Mitchell, A. L. *et al.* InterPro in 2019: improving coverage, classification and access to protein sequence annotations. *Nucleic acids research* **47**, D351-D360, doi:10.1093/nar/gky1100 (2019).

**Figure legends**

**Figure 1. *I. ricinus* SGP*-*YSD library enrichment by human sera. a)** Effect of library enrichment as determined by FACS, showing the percentage of induction induced Xpress-tag positive yeast cells bound by human IgG. The obtained *I. ricinus* SGP*-*YSD was probed with 50 µg/ml IgG from humans with more than 20 reported tick bites per year. (20TB). First 3 sorting rounds were performed by MACS, followed by one time by FACS as shown in b. Columns depict the mean percentage of Xpress and human IgG positive cells, error bars show the standard error of the mean. Western Blot of 10 µg salivary gland extract from 72h fed *I. ricinus* nymph labeled with 20TB human IgG or control IgG is shown in the legend under each respective IgG.  **b)** FACSsort on MACSsort 3. Sort 0, the initial SGP-YSD library and MACSort 3 were induced and labeled with 50 µg/ml 20TB IgG and mouse anti-Xpress IgG followed by anti-human IgG-FITC and anti-mouse IgG-APC. The gate was set on the initial library. Double positive yeast cells were sorted as a whole and as single cell clones for further analysis.

**Figure 2. Validation of identified Tick Salvary Gland Poteins (TSGPs) through FACS analysis of isolated yeast cell clones.** Antibody binding to single yeast cell clones expressing the respective TSGP probed with 50 µg/ml of 20TB IgG or control IgG. When available, multiple clones per TSGP were used. Columns depict the mean percentage of Xpress and human IgG positive cells, error bars show the standard error of the mean.

**Figure 3. Gene expression, recombinant proteins and tick immune rabbit reactivity. a-c)** Gene expression of V5H126, B7PDE7 and A0A0K8R6W3 in nymphal I. ricinus salivary glands (SG). RT-qPCR was performed on B. afzelii infected or uninfected tick SG pools fed for 0, 24 hours and to repletion in triplicates. Columns depict the relative gene expression compared to cytochrome c, error bars show the standard error of the mean. **d)** SDS page of purified recombinant V5H126, B7PDE7 and A0A0K8R6W3. Bands were confirmed as recombinant proteins by anti-HIS IgG (data not shown). **e)** Recombinant protein specific antibody titers in I. scapularis tick immune rabbits. Antigen specific total IgG as measured by ELISA for V5H126, B7PDE7 and A0A0K8R6W3 of rabbits that became tick immune after repeated challenges with nymphal I. scapularis ticks (I. scapularis immune) or normal rabbit serum (NRS). Columns depict means of six replicates and error bars depict Standard Error of the Mean. Significant diffences as determined by multiple t-test with the Holm-Sidak method are indicated by * (P<0,0001).

**Figure 4. The effect of vaccination on tick feeding in cows. a-c)** Antigen specific total IgG reactivity for both cow vaccination experiments as measured by ELISA coated with V5H126, B7PDE7 and A0A0K8R6W3 respectively. The second experiment with the combined vaccination and the control group is indicated with suffix -2 and shown with dashed lines. **d, e)** Tick engorgement weights after single antigen vaccinations for nymphs and adults respectively. The number of engorged nymphs was significantly reduced for V5H126 and A0A0K8R6W3 (P<0.01 and P<0.0001, Chi-Square). Statistical differences in engorgement weights as measured by one-way ANOVA is indicated by * **f,g)** Tick engorgement weights after combined antigen vaccinations for nymphs and adults respectively. Total number of collected engorged ticks are indicated in brackets for each experimental group and was significantly reduced (P<0.01 Chi-Square). Lines indicated means and error bars depict Standard Error of the Mean. Statistical differences as measured by Mann Whitney test is indicated by *.

**Figure 5. Antibody titers, reactivity and tick feeding parameters after vaccination in rabbits.** Antigen specific total IgG endpoint titers as measured by ELISA for V5H126 (**a**), B7PDE7 (**b**) and A0A0K8R6W3 (**c**). Blood was drawn before each vaccination (indicated by arrow) and before tick challenge. End point titers were calculated as Bmin=3*SD. Dots are means and error bars depict Standard Error of the Mean. **d and e**)Tick engorgement weights for nymphs and adults respectively. Total number of collected engorged ticks are indicated in brackets for each experimental group. Lines indicated means and error bars depict Standard Error of the Mean. **f**) Western blot of 10 µg 72 hour fed nymphal and adult salivary gland extract incubated with pooled serum of placebo vaccinated rabbits (left) or V5H126, B7PDE7, A0A0K8R6W3 vaccinated rabbits (right).
