# Supplementary material for: Probing an Ixodes ricinus salivary gland yeast surface display with tick-exposed human sera to identify novel candidates for an anti-tick vaccine
Source: Sci Rep. 2021 Aug 3;11:15745. doi: 10.1038/s41598-021-92538-9 (PMC8333314; doi:10.1038/s41598-021-92538-9)
Supplement: Supplementary file 2 — Supplementary Information 2. [file 41598_2021_92538_MOESM2_ESM.pptx]

## Slide 1
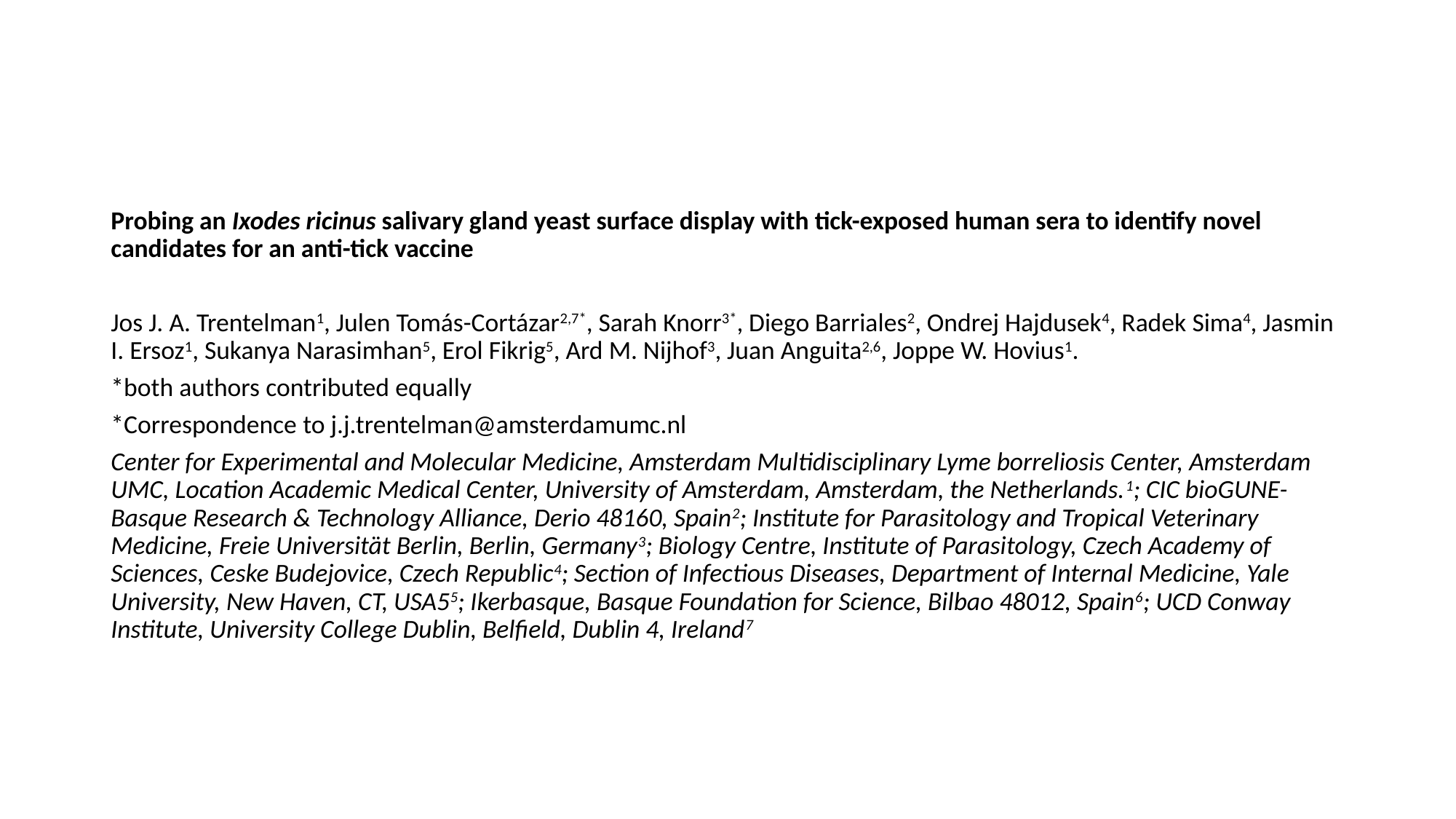

Probing an Ixodes ricinus salivary gland yeast surface display with tick-exposed human sera to identify novel candidates for an anti-tick vaccine
Jos J. A. Trentelman1, Julen Tomás-Cortázar2,7*, Sarah Knorr3*, Diego Barriales2, Ondrej Hajdusek4, Radek Sima4, Jasmin I. Ersoz1, Sukanya Narasimhan5, Erol Fikrig5, Ard M. Nijhof3, Juan Anguita2,6, Joppe W. Hovius1.
*both authors contributed equally
*Correspondence to j.j.trentelman@amsterdamumc.nl
Center for Experimental and Molecular Medicine, Amsterdam Multidisciplinary Lyme borreliosis Center, Amsterdam UMC, Location Academic Medical Center, University of Amsterdam, Amsterdam, the Netherlands.1; CIC bioGUNE-Basque Research & Technology Alliance, Derio 48160, Spain2; Institute for Parasitology and Tropical Veterinary Medicine, Freie Universität Berlin, Berlin, Germany3; Biology Centre, Institute of Parasitology, Czech Academy of Sciences, Ceske Budejovice, Czech Republic4; Section of Infectious Diseases, Department of Internal Medicine, Yale University, New Haven, CT, USA55; Ikerbasque, Basque Foundation for Science, Bilbao 48012, Spain6; UCD Conway Institute, University College Dublin, Belfield, Dublin 4, Ireland7

## Slide 2
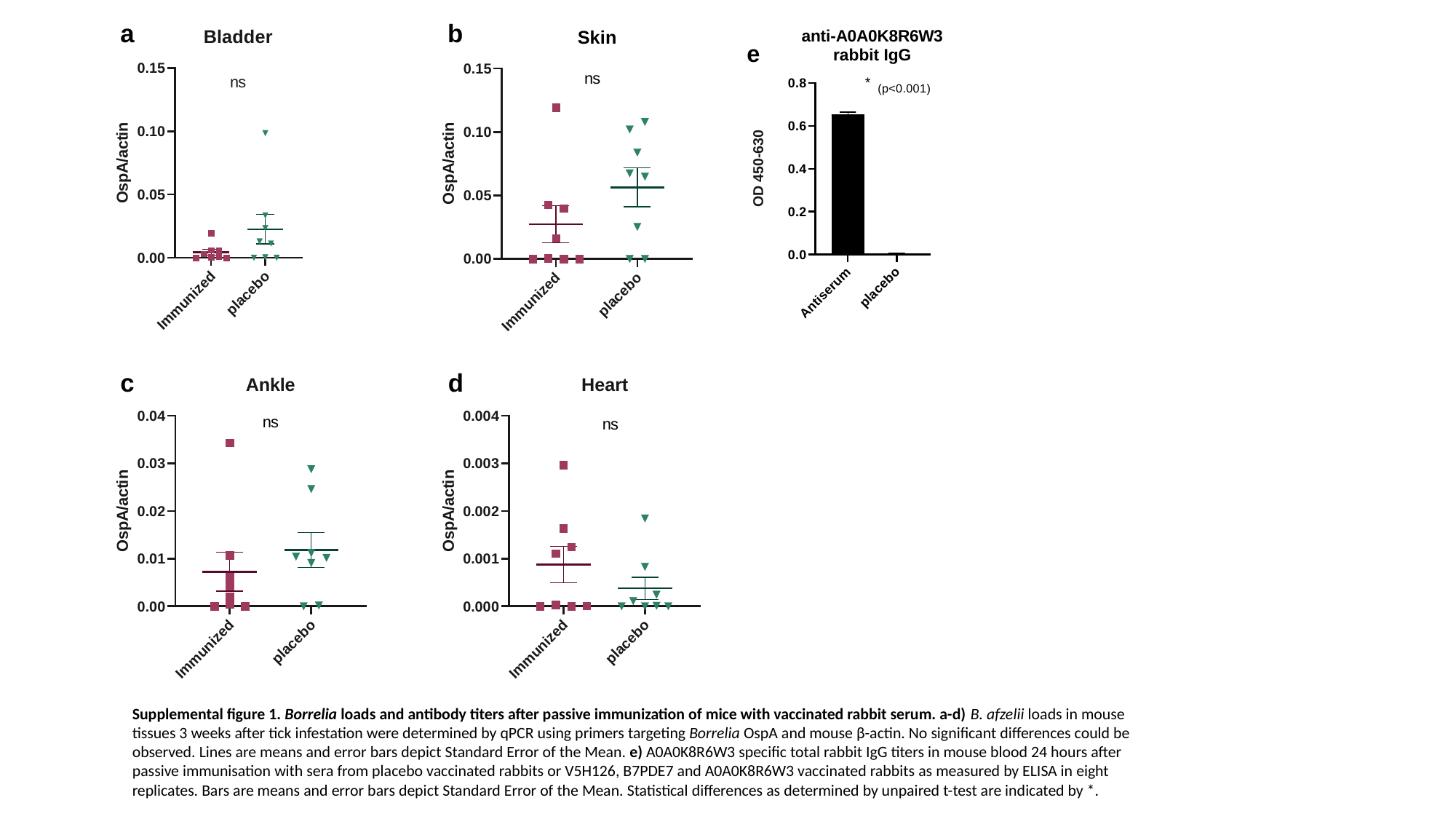

Supplemental figure 1. Borrelia loads and antibody titers after passive immunization of mice with vaccinated rabbit serum. a-d) B. afzelii loads in mouse tissues 3 weeks after tick infestation were determined by qPCR using primers targeting Borrelia OspA and mouse β-actin. No significant differences could be observed. Lines are means and error bars depict Standard Error of the Mean. e) A0A0K8R6W3 specific total rabbit IgG titers in mouse blood 24 hours after passive immunisation with sera from placebo vaccinated rabbits or V5H126, B7PDE7 and A0A0K8R6W3 vaccinated rabbits as measured by ELISA in eight replicates. Bars are means and error bars depict Standard Error of the Mean. Statistical differences as determined by unpaired t-test are indicated by *.

## Slide 3
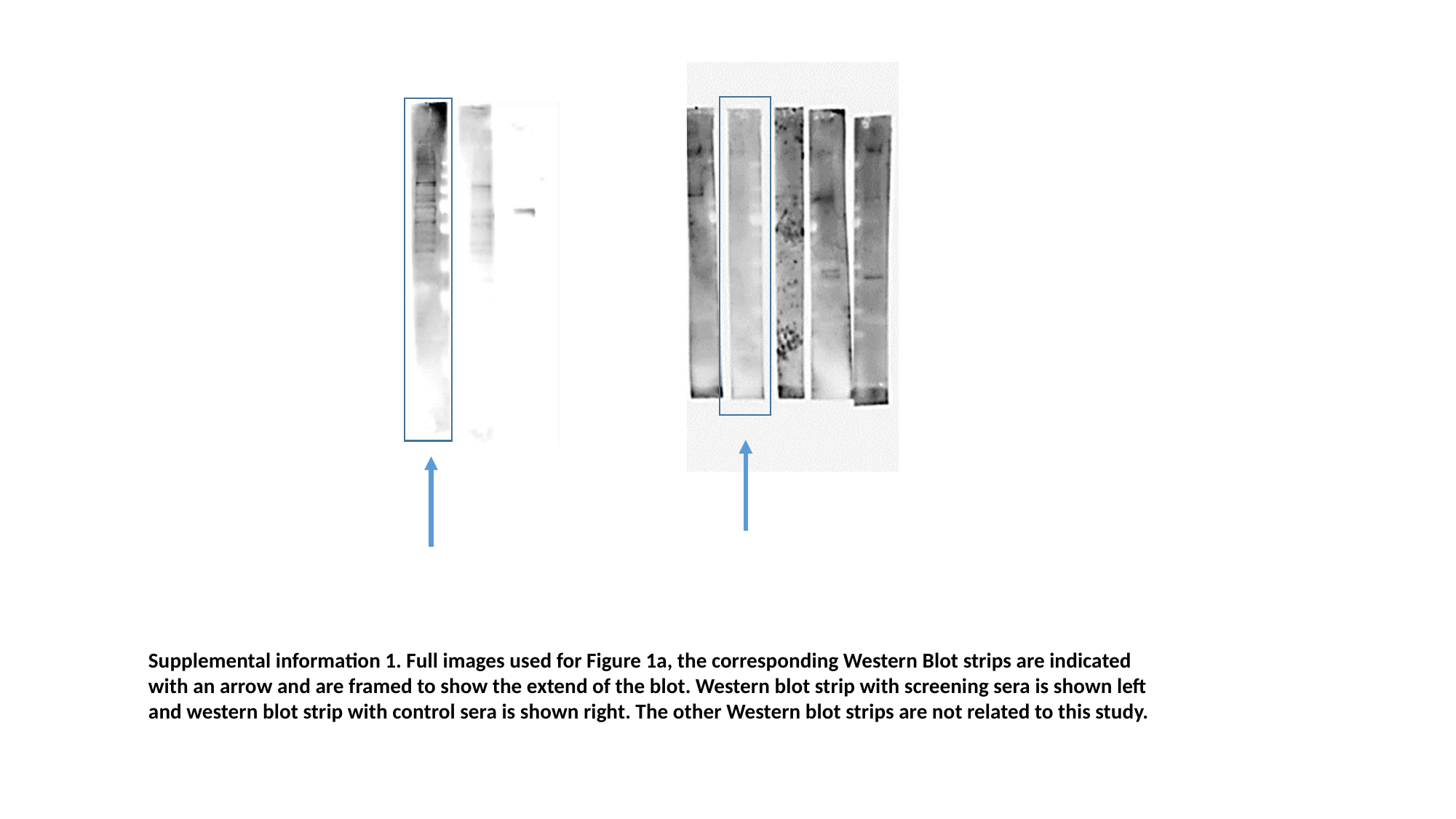

Supplemental information 1. Full images used for Figure 1a, the corresponding Western Blot strips are indicated with an arrow and are framed to show the extend of the blot. Western blot strip with screening sera is shown left and western blot strip with control sera is shown right. The other Western blot strips are not related to this study.

## Slide 4
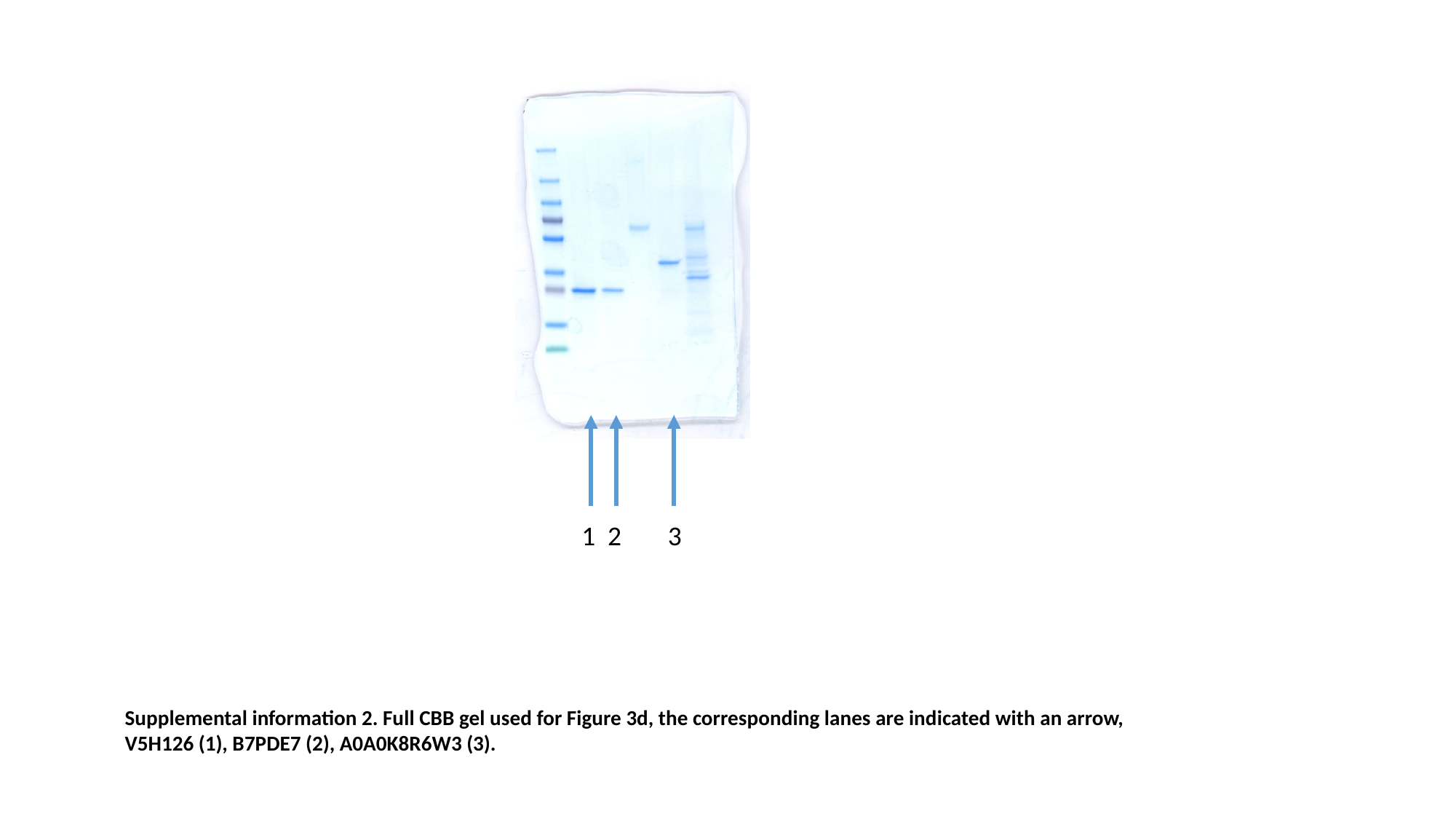

3
1
2
Supplemental information 2. Full CBB gel used for Figure 3d, the corresponding lanes are indicated with an arrow, V5H126 (1), B7PDE7 (2), A0A0K8R6W3 (3).

## Slide 5
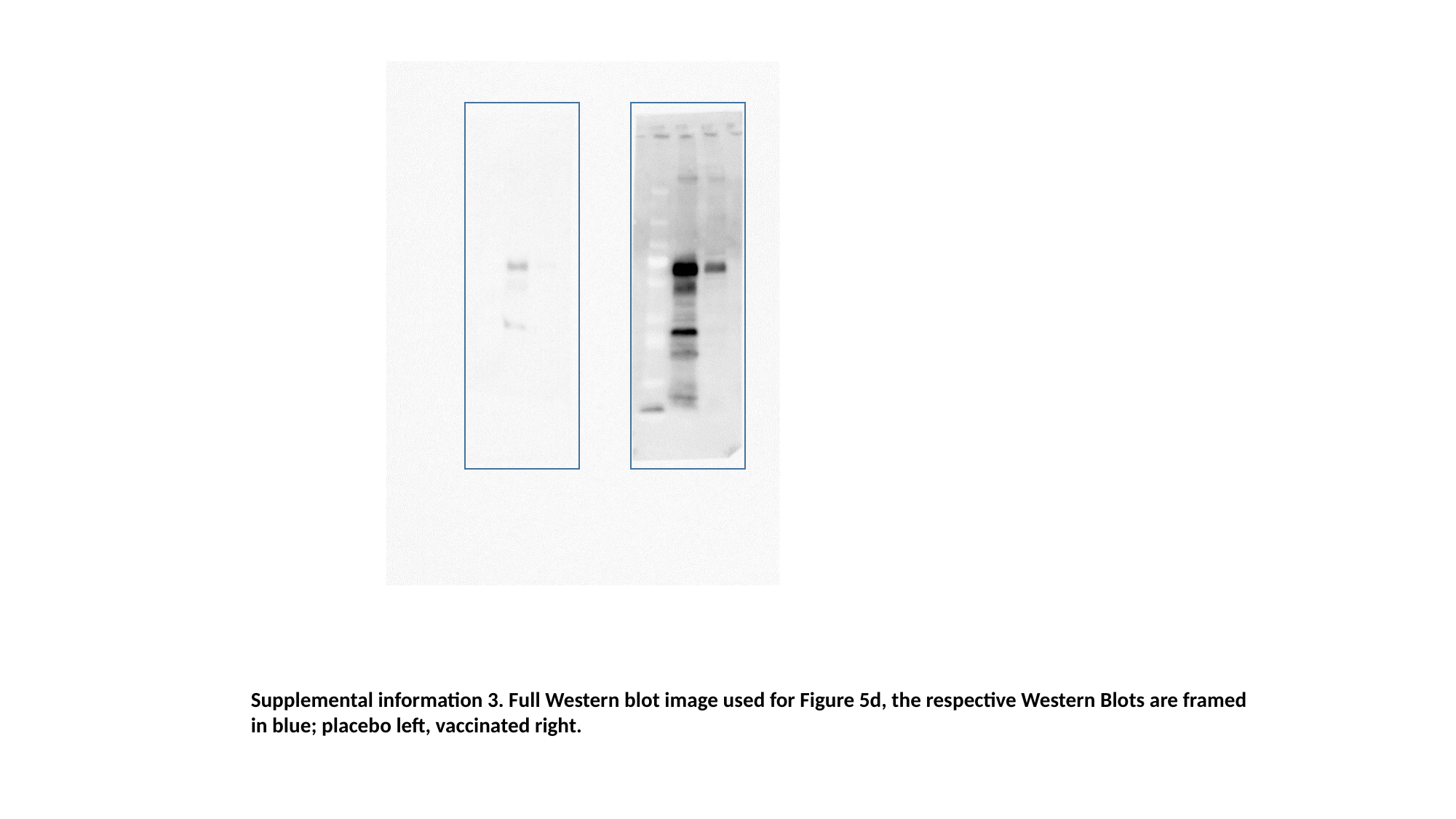

Supplemental information 3. Full Western blot image used for Figure 5d, the respective Western Blots are framed in blue; placebo left, vaccinated right.
